# Shear creep mechanical properties and damage model of mudstone in open-pit coal mine

**DOI:** 10.1038/s41598-022-08488-3

**Published:** 2022-03-25

**Authors:** Guanghe Li, Yanting Wang, Yiming Hu, Dong Wang, Xiaoxu Yang, Yanfei Li, Zhiwei Zhou, Shipeng Zhang

**Affiliations:** 1 College of Mining, Liaoning Technical University, Fuxin, 123000 China; 2 Shenhua Bor Xil Energy Co.Ltd., Hulun Buir, 021008 China

**Keywords:** Civil engineering, Natural hazards

## Abstract

Clarifying the shear creep characteristics of rock and scientifically analyzing its creep deformation law is the key to solving the problem of safe construction and long-term stability analysis of the open-pit coal slope rock mass. Shear creep tests were carried out on mudstone from an open-pit coal mine in Eastern Mongolia to reveal the creep characteristics of mudstone under different normal and shear stresses. Based on the classical Nishihara model, a new composite six-element nonlinear shear creep damage model is established by introducing nonlinear elastomers. Using the least square method, model verification and parameter identification are carried out on the variable test data. At the same time, the influence law of the model parameters on the rock creep deformation is analyzed according to the theoretical curve. The study results showed that the nonlinear shear creeps damage model could better describe the creep characteristics of rock different normal stress and shear stress levels, significantly the non-linear both the strain and time of attenuation creep and accelerated creep. The creep characteristics of the accelerated creep stage described by this model are better than those described by the classical Nishihara model. The model curve is consistent with the changing trend of the experimental data, and the degree of agreement is very high. The correlation coefficients are all above 0.98, which verifies the accuracy and rationality of the model. The influence law of creep parameters is analyzed. The parameters *b* and *c* increase nonlinearly with creep. The increase of *λ* accelerates the process of rock attenuation creep stage; the increase of *η*_10_ slows down the progress of rock decay creep stage; with the increase of *α*, the deformation and creep rate of rock in accelerated creep stage gradually increase. When *η*_2_ increases, the deformation in the acceleration stage decreases gradually. The research results can provide important theoretical support for the safe construction and long-term stability analysis of open-pit coal slope rock masses.

## Introduction

In nature, the failure of rock mass always occurs along the structural plane with relatively weak mechanical properties, and the structure plays a decisive role in the deformation and strength properties of the rock mass to a certain extent^1–5^. Among many deformations and failure of rock mass, shear creep failure is an important part, especially for mudstone slope, the mineral composition is complex, and the strength is low. The instability of such slopes is caused by the long-term deformation superposition, which leads to the final penetration of the main control sliding surface and the landslide^6,7^.

The main lithology of the slopes of most open-pit coal mines in Eastern Mongolia is mudstone, with a buried depth of 5–160 m and low strength. Creep characteristics are very significant under the action of the Neogene, Paleogene, and other overlying rock loads and the long-term self-gravity load. Once the long-term shear strength or limit equilibrium state is reached, it is easy to induce the failure of the main control structural plane^8–11^. Therefore, it is necessary to further study the shear creep damage model of mudstone in open-pit mines, which provides a reference for revealing the creep characteristics of mudstone slopes, and has important theoretical significance and practical value for the construction of open-pit rock mass and the long-term stability of slopes.

Many experts and scholars have done a lot of meaningful research work on the shear creep model. Chen et al.^12^ carried out nuclear magnetic resonance (NMR) detection and shear creep test of red sandstone with different moisture content after freeze–thaw, revealed the influence mechanism of freeze–thaw cycle and moisture content change on the microstructure and creep characteristics of red sandstone, and established the freeze–thaw shear creep model of red sandstone. Han et al.^13^ conducted the shear creep test on the original specimen of the interlayer staggered zone, and established the unsteady visco-elastoplastic creep model based on the element method and the yield surface creep model to reveal the creep characteristics, creep rate, and long-term strength of the interlayer staggered zone. Shi et al.^14^ carried out a series of direct shear tests on different rocks under different normal stress conditions to study the shear mechanical properties and macro–micro fracture characteristics of the rock. Rock failure properties include plastic failure, brittle characteristics, and ductility characteristics. Zhang et al.^15^ studied the shear mechanical properties of mudstone and weak interlayer in the Tianshui area of Gansu Province. The weak layer samples showed strain-softening characteristics, and the shear failure occurred within the weak layer. The microscopic characteristics and shear creep tests of granite samples subjected to different freeze–thaw cycles were carried out by Zhang et al.^8^. The results show that with the increase of freeze–thaw cycles, the rock surface damage is more obvious, the creep deformation and creep rate are gradually increased, and the failure stress and long-term strength are decreased. Song et al.^16^ carried out the graded shear creep test of rock mass with anchor joints under different normal stresses. The GTN model was introduced to modify the traditional Nishihara model, which can improve the accurate description of the accelerated creep stage of rock and obtain the composite rheological model to describe the overall shear creep of rock mass with anchor joints. Liu et al.^17^ established the creep constitutive model of mica schist by indoor shear creep test, introducing the four-element linear viscoelastic Burgers model and the improved Burgers model, and used the two models to fit the whole process curve of shear creep to obtain the model parameters. The correctness and rationality of the model were verified by comparative analysis. Yu et al.^18^ carried out the direct shear test on the soft rock under different moisture content. According to the literature: The traditional Kelvin model, Nishihara and Burgers creep models can describe the deformation characteristics of rock attenuation creep and constant velocity creep. However, they cannot describe the nonlinear law of strain and time in the accelerated creep stage. Many literatures have established creep models under different lithologies, disturbances and environments, which have laid a theoretical foundation for this research. However, there are few studies on the creep characteristics of open-pit coal mine mudstone under low-stress state, and no unified creep model and creep equation have been established. Therefore, the purpose of this study is to understand the shear creep characteristics and laws of mudstone comprehensively and deeply. Based on the classical Nishihara model, a new six-element nonlinear shear creep damage model of composite materials was established by introducing nonlinear elastic elements and connecting elastic body, variable parameter viscoelastic body, and damage viscoplastic body in series. The least-square method is used for model verification and parameter identification of the variable test data. At the same time, the influence of model parameters on rock creep deformation is analyzed according to the theoretical curve.

## Direct shear creep tests of mudstone

### On-site sampling and sample preparation

**Figure 1:**
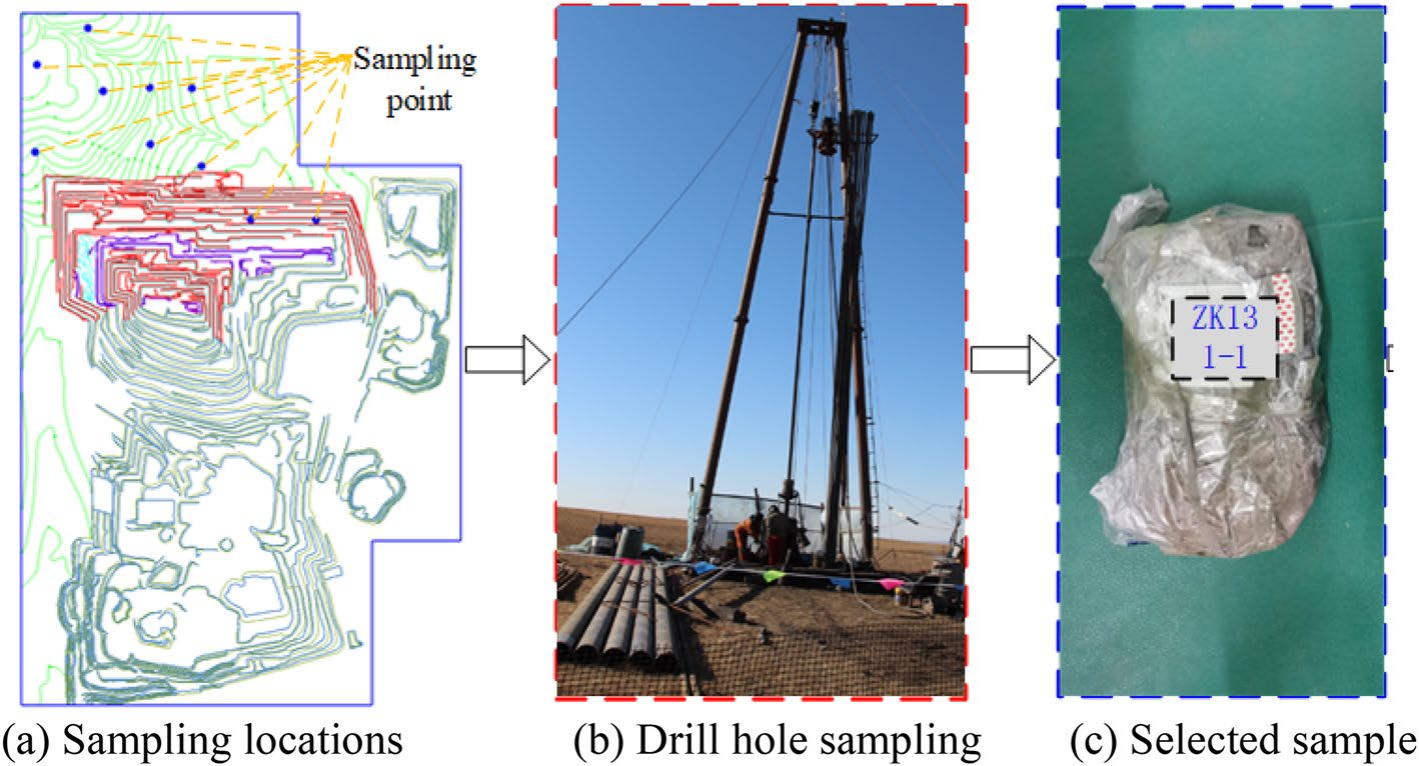
Standard rock sample preparation process.

The test samples come from mudstone above the roof of coal 1 in an open-pit coal mine in Eastern Mongolia. The rock samples are mainly obtained through geological engineering drilling, with a hole diameter of 85 mm. The rock samples are packaged and sealed with double-layer plastic bags and labeled on them. In the laboratory, coat the inner wall of the ring cutter with a layer of petroleum jelly, press it on the drilled rock sample, and cut while pressing until the soil sample protrudes above the ring cutter, and then flatten both ends of the ring cutter to make a standard direct shear creep sample. The sample size is *ϕ*61.8 mm × 20 mm cylinder. The specific process is shown in Fig. 1.

### Test equipment and test scheme

**Figure 2:**
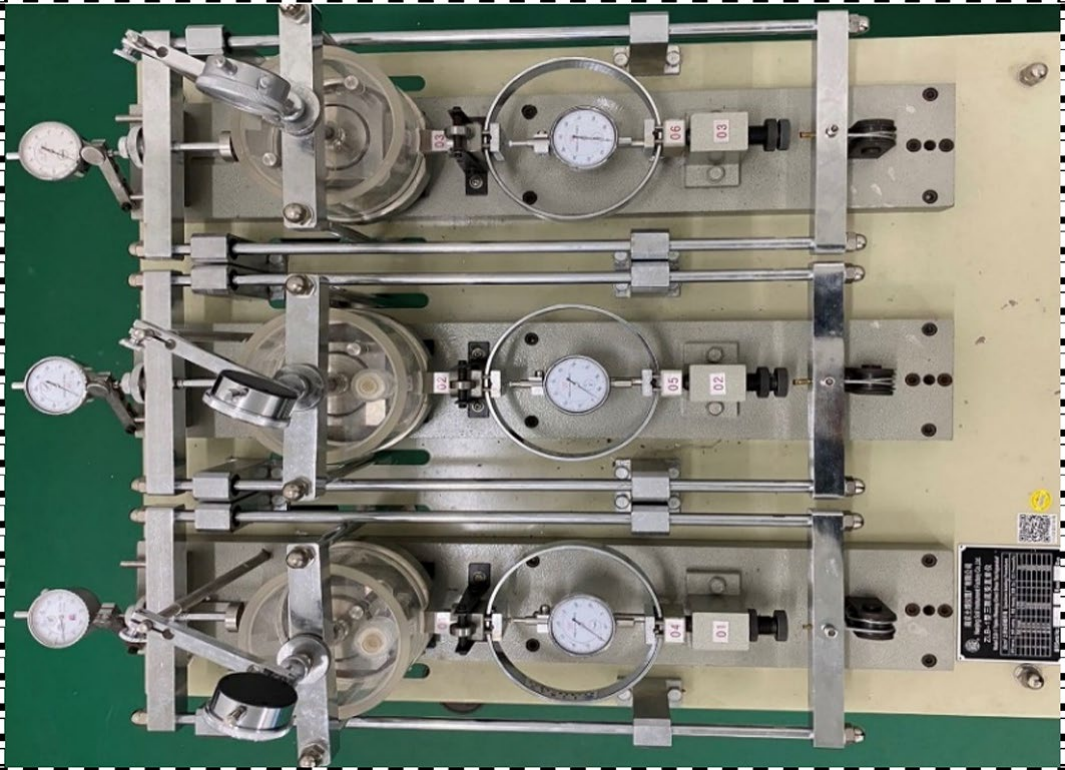
The ZLB-1 direct shear creep testing machine.

The ZLB-1 direct shear creep testing machine is used as the test equipment. This equipment is mainly composed of a lever for loading vertical load and shear load, a shear box, a force ring, an instrument for measuring vertical creep strain and shear creep strain, and a plexiglass humidifying box. The maximum vertical load is 600 kPa and the maximum shear load is also 600 kPa. The equipment is shown in Fig. 2.

Take out the rock sample from the humidifier and perform the fast shear test. The fast shear strength and the shear stress failure value *τ*_p_ of different normal stress levels is tested. The test results are shown in Table 1.

**Table 1:** The shear strength under different normal stresses.

Normal stress (kPa)	Shear strength (kPa)
100	72
200	136
300	168

**Figure 3:**
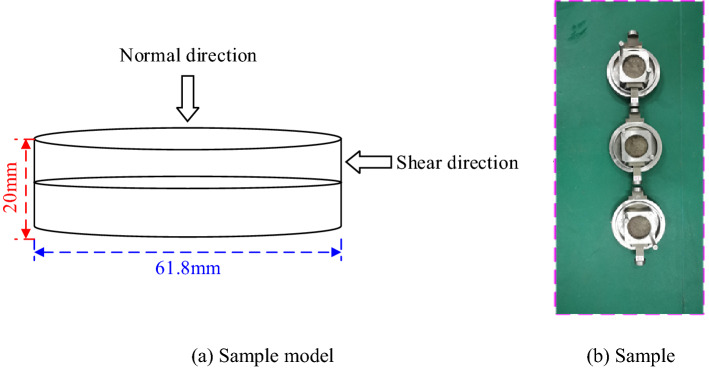
Shear creep diagram.

A shear creep test was performed on the specimen. During the test, the sample is placed between the upper and lower shear boxes, and the lower shear box is fixed on the horizontal guide rail of the testing machine. The shear load is applied to the upper shear box through the shear loading system, and at this time, the lower shear box is also subjected to small equal and opposite forces through the counter-force device. The normal load is applied to the upper surface of the upper shear box through the normal loading system. The central axis of the sample in the vertical direction coincides with the central axis of the normal indenter, and the central axis of the sample in the horizontal direction is in the middle of the upper and lower shear boxes. Figure 3 is a schematic diagram of sample shear creep.

According to the shear strength results under different normal stresses, the four-stage stress loading scheme is set according to the shear strength of 0.5/0.6/0.7/0.8 times under each normal stress, and the shear creep test is carried out. When the shear deformation rate is less than 5 × 10^-4^ mm/d, the next level of shear load is applied until the specimen is sheared. The test plan is shown in Table 2.

**Table 2:** Hierarchical loading scheme.

Normal stress (kPa)	The shear stress is loaded step by step
100	36 kPa→43.2 kPa→50.4 kPa→57.6 kPa
200	68 kPa→81.6 kPa→95.2 kPa→108.8 kPa
300	84 kPa→100.8 kPa→117.6 kPa→134.4 kPa

## Analysis of test results

**Figure 4:**
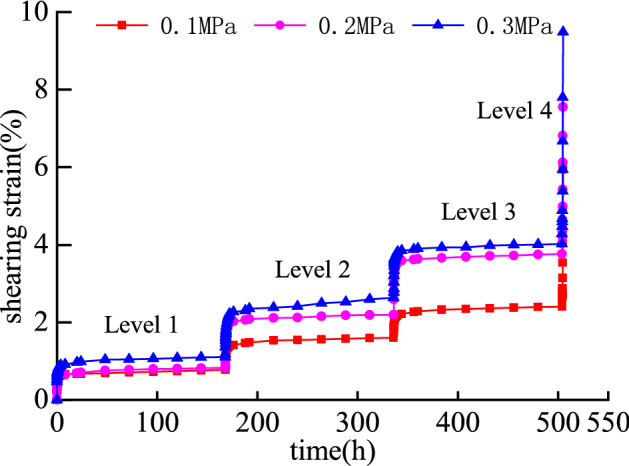
Mudstone creep curve under step loading.

According to the shear creep test, the shear creep curve of the mudstone sample under different normal stress and shear stress is shown in Fig. 4.

**Figure 5:**
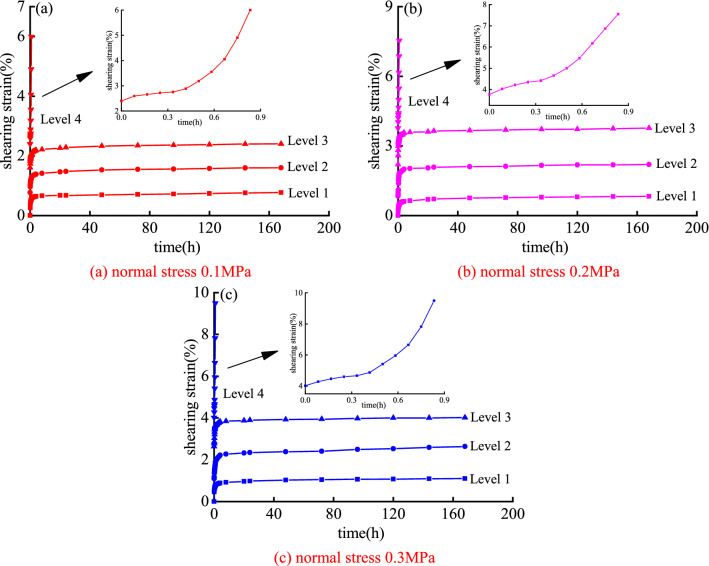
Mudstone creep curve under different normal stress.

Using the Boltzmann superposition principle, the creep curves under the staged loading conditions are transformed, and the creep curves under the separate loading conditions are obtained in Fig. 5.

Figure 5 shows that when the shear stress level is from the first level to the third level, the mudstone sample generates instantaneous elastic strain in the initial stage of loading stress. However, as the loading time increases, the shear strain rate of the specimen gradually decreases, and the shear strain increases non-linearly, showing the characteristics of attenuated creep. After that, the shear strain tends to a constant value and remains stable for a long time, and the constant velocity creep becomes the main of the entire creep process. When the mudstone specimen is loaded with the fourth-level shear stress, the displacement of the specimen undergoes a short-term attenuation and creep, which has a non-linear accelerated creep stage. In summary, the creep stages of mudstone samples include: attenuation creep, constant velocity creep, and accelerated creep stages.

## Establishment of the nonlinear shear creep damage model

**Figure 6:**
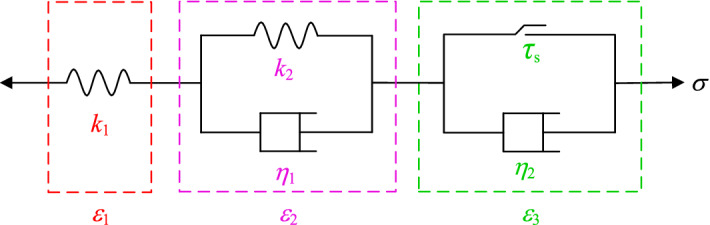
Nishihara model.

**Figure 7:**
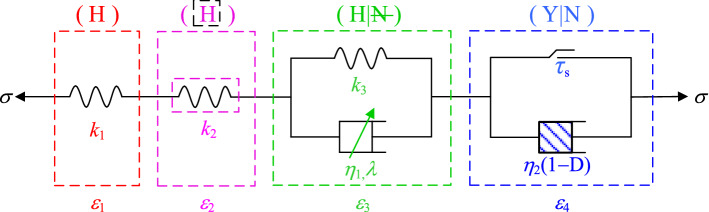
Nonlinear shear creep damage model.

The classic Nishihara model can better describe the attenuation of the rock creep process and the deformation characteristics of the constant-velocity creep stage. However, since the components used in the Nishihara model are ideal linear components, it is difficult to describe the nonlinear deformation law of the rock in the accelerated creep stage^19–21^. However, the introduction of creep damage in the accelerated creep stage can better describe the full creep process of the rock^22–24^. Therefore, based on the Nishihara model shown in Fig. 6, the author introduced a nonlinear elastomer and damage variable *D* and established a new nonlinear shear creep damage model as shown in Fig. 7.

### Elastomer

Liu et al.^25^ established a creep equation of an elastomer,

1 $$\varepsilon_{1} = \frac{\tau }{{k_{1} }}$$

where *τ* is the shear stress of the elastomer, *k*_1_ is the elastic modulus of the elastomer, and *ε*_1_ is the strain of the elastomer.

### Nonlinear elastomer

Wang et al.^26^ defined a new nonlinear function as follows:

2 $$f(t) = \frac{{bt^{c} }}{{1 + bt^{c} }}$$

where *t* is the independent variable, *f*(t) is the dependent variable, *b* and *c* are parameters greater than 0.

When *b* and *c* take an appropriate value, the relationship between *t* and *f*(*t*) is: when *t* → 0, *f*(*t*) → 0; when *t* → ∞, *f*(*t*) → 1. And *f*(*t*) is a continuously increasing function, when the rock creeps time increases to a certain extent, *f*(*t*) approaches 1, so it is assumed that *f*(*t*) = 1 after *t* reaches a constant time.

Under constant stress, it is assumed that the attenuation law of elastic coefficient of nonlinear elastomer satisfies^27^:

3 $$k_{2} = \frac{{k_{20} }}{f(t)}$$

where *k*_20_ is the initial value of the elastic modulus of the nonlinear elastomer, *k*_2_ is the elastic modulus of the nonlinear elastomer.

Nonlinear elastomer’s constitutive equation is

4 $$\varepsilon_{2} = \frac{\tau }{{k_{2} }}$$

where *τ* is the shear stress of the nonlinear elastomer, *k*_2_ is the elastic modulus of the nonlinear elastomer, and *ε*_2_ is the strain of the nonlinear elastomer.The creep equation of the nonlinear elastomer is established as follows:

5 $$\varepsilon_{2} = \frac{\tau }{{k_{2} }} = \frac{\tau f(t)}{{k_{20} }} = \frac{{\tau bt^{c} }}{{k_{20} (1 + bt^{c} )}}$$

#### Varying-parameter viscoelastic body

Zhang et al.^28^ assumed that the viscosity coefficient is a power function related to time; function *η*_1_(t) of the viscosity coefficient related to time is given as follows:

6 $$\eta_{1} (t) = \eta_{10} t^{1 - \lambda }$$

where *η*_10_ is the initial viscosity coefficient of the varying-parameter viscoelastic body, and λ is a constant.

According to the constitutive equation of Kelvin’s material, the creep equation of the varying-parameter viscoelastic body is established as follows^27^:

7 $$\tau = k_{3} \varepsilon_{3} + \eta_{1} (t)\dot{\varepsilon }_{3} = k_{3} \varepsilon_{3} + \eta_{{{10}}} t^{1 - \lambda } \dot{\varepsilon }_{3}$$

where *τ* is the shear stress of the varying-parameter viscoelastic body, *ε*_3_ is the strain of the varying-parameter viscoelastic body, and *k*_3_ is the elastic modulus.

The creep equation of the varying-parameter viscoelastic body is established by integrating Eq. (7) as follows:

8 $$\varepsilon_{3} = \frac{\tau }{{k_{3} }}\left[ {1 - \exp ( - \frac{{k_{3} }}{{\eta_{{{10}}} \lambda }}t^{\lambda } )} \right]$$

#### *Damaged viscoplastic body *

The author introduces the damage variable *D* to describe the creep damage degradation of the viscosity coefficient and constructs a viscoplastic body considering the damage. Its constitutive equation can be written as follows^29^:

9 $$\tau = \eta_{2} (t)\dot{\varepsilon }_{4}$$

where *τ* is the shear stress of the damaged viscoplastic body,$\dot{\varepsilon }_{4}$ is the strain rate of the damaged viscoplastic body, and *η*_2_(*t*) is the function of the viscosity coefficient related to time.

Based on the results of numerous rock creep damage tests, the damage variable *D* took the form of a negative exponential function related to time during rock creep^30–35^. In this study, the damage variable is expressed as Eq. (10).

10 $$D = 1 - \exp ( - \alpha t),(0 < D < 1),$$

where *α* is the coefficient related to the properties of rock materials.

Based on $\eta_{2} (t) = \eta_{2} (1 - D) = \eta_{2} \exp ( - \alpha t)$ and Eq. (9), the creep equation of the damaged viscoplastic body can be written as follows:

11 $$\varepsilon_{4} = \frac{\tau }{{\eta_{2} \alpha }}\exp (\alpha t),$$

where *ε*_4_ is the strain of the damaged viscoplastic body, and *η*_2_ is the initial viscosity coefficient of the damaged viscoplastic body.

### Establishment of the nonlinear shear creep damage model

According to the series character of components, the following relationship can be obtained^36,37^:

12 $$\varepsilon = \varepsilon_{1} + \varepsilon_{2} + \varepsilon_{3} + \varepsilon_{4}$$

Based on Eq. (1), Eq. (5), Eq. (8), Eq. (11), and Eq. (12), the creep equation of the nonlinear shear creep damage model can be obtained as

13 $$\left\{ {\begin{array}{*{20}l} {\varepsilon = \frac{\tau }{{k_{1} }} + \frac{{\tau bt^{c} }}{{k_{20} (1 + bt^{c} )}} + \frac{\tau }{{k_{3} }}\left[ {1 - \exp ( - \frac{{k_{3} }}{{\eta_{{{10}}} \lambda }}t^{\lambda } )} \right]} \hfill & {\tau < \tau_{s} } \hfill \\ {\varepsilon = \frac{\tau }{{k_{1} }} + \frac{{\tau bt^{c} }}{{k_{20} (1 + bt^{c} )}} + \frac{\tau }{{k_{3} }}\left[ {1 - \exp ( - \frac{{k_{3} }}{{\eta_{{{10}}} \lambda }}t^{\lambda } )} \right] + \frac{{\tau - \tau_{s} }}{{\eta_{2} \alpha }}\exp (\alpha t)} \hfill & {\tau \ge \tau_{s} } \hfill \\ \end{array} } \right.$$

where *τ*_*s*_ is the yield strength of a rock.

## Model parameter identification and influencing factors analysis

### Model parameter identification

**Figure 8:**
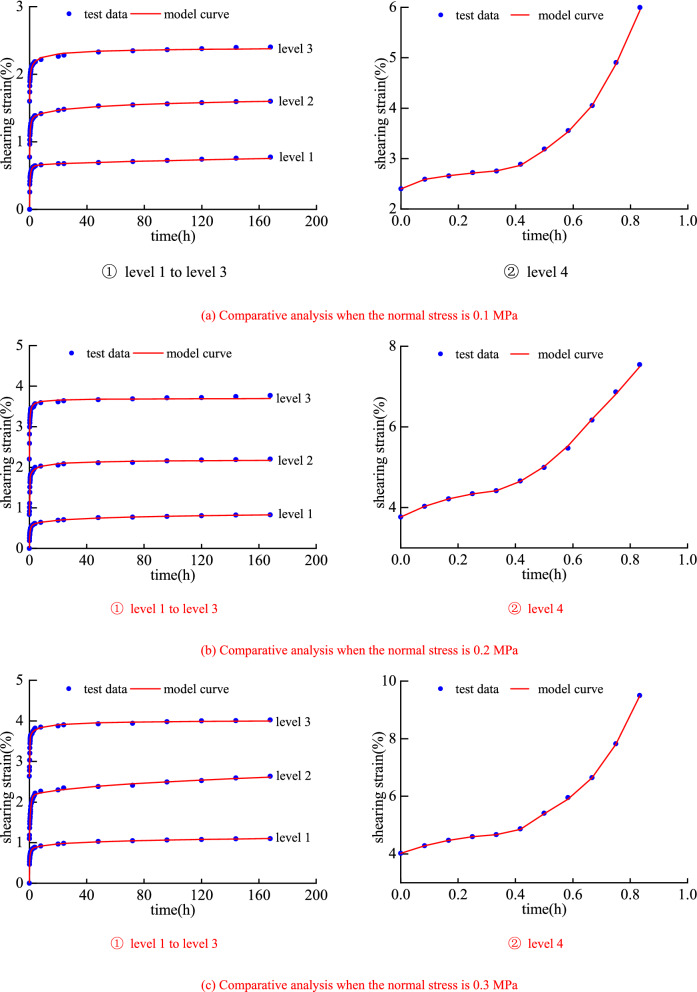
Comparative analysis between the model curve and the test data.

**Figure 9:**
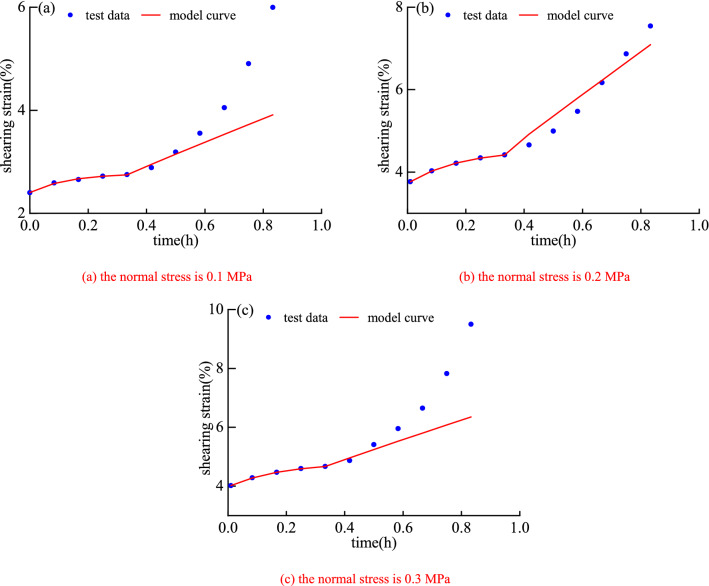
Comparative analysis between the Nishihara model curve and the test data.

Based on the results of the shear creep test, the identification of model parameters with a reasonable method is an indispensable part of the creep model research. Based on MATLAB, the author uses the least square method to identify the creep parameters^38–41^. The parameter identification results are shown in Table 3, and the comparison between the model curve and the experimental data is shown in Fig. 8. The comparison between the model in this paper and the classical Nishihara model is shown in Fig. 9.

**Table 3:** Model parameter identification results.

Normal stress	Shear stress	*k*_1_	*k*_20_	*k*_3_	*λ*	*b*	*c*	*η*_10_	*α*	*η*_2_	*R*^2^
/MPa	/MPa	/MPa	/MPa	/MPa				/MPa·h		/MPa·h	
0.1	0.0360	1.8419	0.0021	0.0596	0.5716	0.0019	0.3268	0.0412	–	–	0.9977
	0.0432	0.9902	0.0157	0.1196	0.0427	3.8030	1.4025	0.9206	–	–	0.9997
	0.0504	0.5180	0.0536	0.3324	0.0261	1.7567	0.4791	0.3892	–	–	0.9993
	0.0576	0.0240	0.0996	1.0947	0.8054	1.3319	0.2110	0.0724	1.7113	0.0031	0.9833
0.2	0.0680	4.1770	0.0135	0.0138	0.6779	0.1348	0.5000	0.1024	–	–	0.9995
	0.0816	2.1658	0.0860	0.1276	0.0433	8.7609	2.1109	1.7892	–	–	0.9982
	0.0952	1.8412	0.0992	0.6375	0.0255	9.6436	1.5091	1.0956	–	–	0.9964
	0.1088	0.0293	0.2538	1.5919	1.1940	1.8282	0.2492	0.0183	1.1442	0.0041	0.9951
0.3	0.0840	5.0613	0.0153	0.0233	0.7505	0.0877	0.1073	0.1526	–	–	0.9997
	0.1008	2.2110	0.0988	0.1289	1.3513	0.0013	0.0590	0.0755	–	–	0.9975
	0.1176	1.8013	0.1399	0.2327	0.0189	11.6287	2.3420	2.1062	–	–	0.9989
	0.1344	0.0332	0.2936	1.9347	1.2444	1.6682	0.1902	0.0195	1.5250	0.0043	0.9832

It can be seen from Fig. 8 that the nonlinear shear creep damage model can better characterize the creep characteristics of mudstone, especially the nonlinear change law of strain and time in the accelerated creep stage. Moreover, a certain error occurs between the model curve and the test data, indicating that the model can achieve results under different normal stress and shear stress levels. The better fit results demonstrate the correctness and applicability of the model.

As shown in graphs in Fig. 8 under level 1 to level 3, as one can see that the shear strain gradually increases with time and tends to a certain value. The model curve is consistent with the experimental data trend, and the agreement between the two is relatively high. Hence, the model is also suitable for studying the law of stable creep deformation of rocks.

It can be observed from graphs in Fig. 8 under level 4 that in the accelerated creep stage, the shear strain increases in a "concave" shape and nonlinearly with time, and the model curve is consistent with the experimental data, especially the description of the inflection point is more accurate. It shows that the model can accurately capture the law of accelerated creep deformation of mudstone, which fully reflects the superiority of the established model for describing the nonlinear accelerated creep characteristics of the rock.

Overall, the nonlinear shear creep damage model has a better identification effect and a higher fitting accuracy under different normal stress and shear stress. The fitting curve is highly consistent with the experimental data, the correlation coefficient *R*^2^ is greater than 0.98. It shows that the model constructed in this paper can accurately describe the long-term shear creep characteristics of mudstone under different normal stress and shear stress. The research results can provide important theoretical support for the safe construction and long-term stability analysis of open-pit coal slope rock masses.

It can be seen from Fig. 9 that the fitting curve of the Nishihara model can better characterize the attenuation of rock creep and the creep law of the constant creep stage, but it cannot well describe the creep characteristics of the accelerated creep stage of rock. The rock shear creep model constructed in this paper can better characterize the creep characteristics of rock in the accelerated creep stage, which is the advantage of this model over the Nishihara model.

### Influencing factors analysis

**Figure 10:**
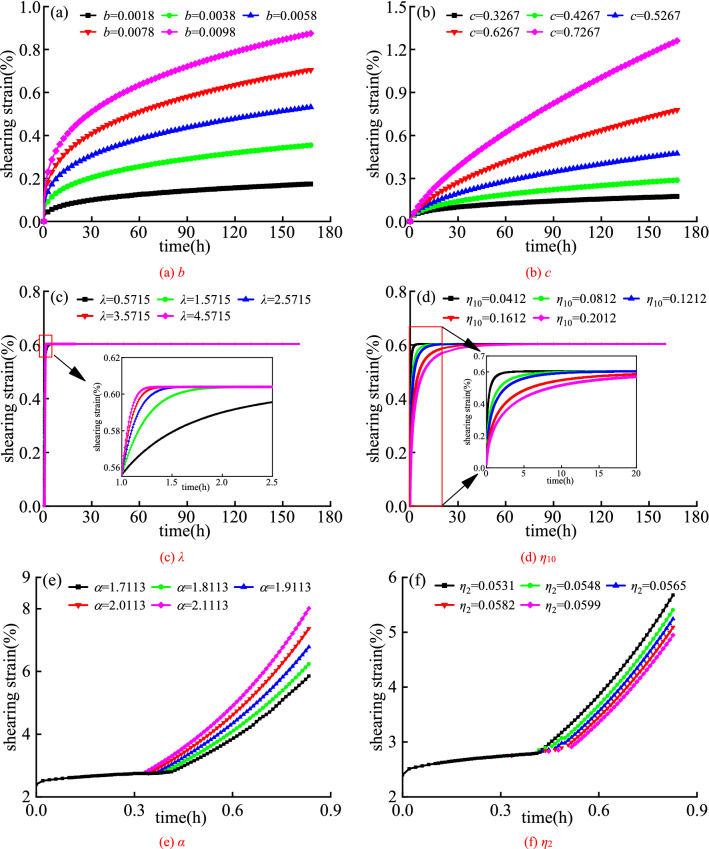
Creep curves of rock with different b, c, *λ*, *η*_10_, α and *η*_2_.

Based on the test data of 0.1 MPa normal stress and 0.0360 MPa shear stress, Fig. 10a,b shows the influence of different values of parameters b and c on the creep curve of nonlinear elastomer, and Fig. 10c,d shows the effect of different values of parameters d and e on the varying-parameter viscoelastic body. Based on the test data of 0.1 MPa normal stress and 0.0576 MPa shear stress, Fig. 10e,f shows the influence of different values of parameters b and c on the creep curve of the damaged viscoplastic body.

Figure 10a,b indicates that the amount of creep deformation of the rock with the increase of the parameters *b* and *c* at the same time. But compared with Fig. 10a, with the increase of *c*, the creep rate of rock increases faster and enters the stage of constant velocity creep faster in Fig. 10b. The model curve in Fig. 10a,b has obvious attenuated creep deformation, which further illustrates that it is feasible to use defined nonlinear functions and nonlinear elastomer elements to simulate rock attenuated creep deformation.

It can be seen from Fig. 10c,d that the larger the *λ* is, the shorter the time required for the rock from decay creep to constant creep is, and the faster the creep slope decays in decay creep. The greater the *η*_10_, the longer the time required for rock from decay creep to constant creep, the slower the decay rate of creep slope in the decay creep stage. It shows that the model can reflect the characteristics of rock creep curves under different creep times and different stress states, and its applicability is more extensive than that of the traditional Kelvin model.

It can be seen from Fig. 10e,f that when other parameters remain unchanged, with the increase of *α*, the steady-state time of the rock gradually decreases, and the accelerated creep rate and creep deformation gradually increase, and the rock is more likely to become viscoelastic transition to viscoplasticity. Keeping other parameters unchanged, as the viscosity coefficient *η*_2_ increases, the steady-state creep time of the rock increases, and the creep deformation of the acceleration section gradually decreases. The above two points are the influence law of the parameters and the viscosity coefficient on the strain in the accelerated creep stage, which reflect the correctness and applicability of the established damaged viscoplastic body.

### Next step of research

In this paper, through the shear creep test of mudstone, the shear creep model is constructed to explore the creep characteristics and deformation law of mudstone in the whole stage. In the actual engineering of the open-pit coal mine, groundwater and atmospheric precipitation go deep into the slope, which is an important factor affecting the stability of the mudstone slope. To enhance the applicability of the model, the creep deformation characteristics and constitutive model of mudstone under different water contents will be studied in one step.

## Conclusion

Based on the classical Nishihara model, a nonlinear elastic body was introduced, and a composite six-element nonlinear shear creep damage model including elastic body, nonlinear elastic body, variable parameter viscoelastic body, and viscoplastic body with damage considered was constructed. Taking the mudstone of the Inner Mongolia open-pit coal mine as the experimental object, the shear creep test under different normal stresses was carried out, and the parameters of the test data were identified.

1. The creep characteristics of the accelerated creep stage described by this model are better than those described by the classical Nishihara model.
2. The fitting curve is highly consistent with the experimental data, the correlation coefficient *R*^2^ is more than 0.98. It shows that the model constructed in this paper can accurately describe the long-term shear creep characteristics of mudstone under different normal stress and shear stress.
3. The influence law of creep parameters is analyzed. The parameters *b* and *c* increase nonlinearly with creep. The increase of *λ* accelerates the process of rock attenuation creep stage; the increase of *η*_10_ slows down the progress of rock decay creep stage; with the increase of *α*, the deformation and creep rate of rock in accelerated creep stage gradually increase. When *η*_2_ increases, the deformation in the acceleration stage decreases gradually.
4. The research results can provide important theoretical support for the safe construction and long-term stability analysis of open-pit coal slope rock masses.

## Data Availability

The data used to support the findings of this study are available from the corresponding author upon request.
